# Supplementary material for: The Simpsons did it: Exploring the film trope space and its large scale structure
Source: PLoS One. 2021 Mar 31;16(3):e0248881. doi: 10.1371/journal.pone.0248881 (PMC8011780; doi:10.1371/journal.pone.0248881)
Supplement: Attachment — Submitted filename: TROPES_v2.pdf [file pone.0248881.s001.pdf]

Dear Editor,

We would like to express our gratitude to the editorial team and the reviewers, whose valuable comments and suggestions are highly appreciated and have been followed in this new version of the paper. The main differences with respect to the previous version are:

- The abstract has been shortened.
- The motivation of the paper in the introduction has been clarified.
- New references have been added to improve the state of the art.
- Some concepts have been explained in more depth.
- Some decisions in the design of the methodology have been justified.
- Conclusions have been improved.
- The format of some figures has been changed and the font of the tables has been increased, as well as the correction of several grammatical errors.

Please find below a detailed description of the changes we have made in the manuscript to address the reviewers' comments. All modifications in the manuscript are highlighted in blue, including the changes after language correction.

Best regards.
The authors.

---

# 1 Reviewer #1

**Legend:**

RV: Reviewer.

AA: Author answers.

---

**RV:**

This paper provides insights in understanding the tropes that compose the films. The authors study data from TV Tropes and IMDb websites using scientometric and complex networks methods.

The dataset is interesting and well described. The paper has a proper introduction into the research questions. However, there are a number of things regarding the description of methodology that could be improved. Below are comments that could help in making some sections more clear to a reader

**AA:**

We want to thank the reviewer for the kind comments.

**RV:**

1. The authors often use "co-words network" and "co-occurence network" as metaphors to "co-tropes" or "co-films" network. This could be misleading and confusing, e.g. Algorithm 1, "Fig. 8 shows the spanning tree of the co-word network", ...

**AA:**

The reviewer is right. We have updated the references to "co-word" to "co-trope" or "co-film" instead, so that the document is easier to understand and better fits the network we are describing.

**RV:**

2. The authors use Jaccard index to compare tropes between two genres. The explanation "the Jaccard index is computed for each set of tropes within the genres in respect to the rest of genres tropes." is not precise.

**AA:**

We agree with the reviewer, the sentence is not precise and could lead to confusion. For each genre we gather the tropes that appear in the films of that genre. The Jaccard index is computed for each possible pair of two sets of tropes. Hence, to compare the genres, the Jaccard index is computed for each possible pair of set of tropes, each of them corresponds to a genre. To clarify this sentence, we have rewritten it as follows (line 295, page 7):

> *the Jaccard index is computed for each possible pair of set of tropes, each of them corresponding to a genre.*

**RV:**

3. The authors should define difference between community and cluster. Sometimes, it seems that authors use these two terms as synonyms. However, in l288, p8 they write: "This means that a film can belong to more than one cluster for the same community."

**AA:**

We thank the reviewer for pointing this out. Indeed, community is a synonym for cluster in our manuscript. We should have written "node" instead of cluster. Hence, the sentence has been rewritten (line 333, page 9):

> *This means that a film can belong to more than one node for the same community.*

Moreover, we have unified "cluster" and "community" in the rest of the text to avoid confusion.

---

**RV:**

4. The authors should provide exact definition of density, centrality and central trope.

---

**AA:**

We agree with the reviewer, therefore we have rewritten the visualization explanation including the definition of density and centrality. Moreover we have detailed the meaning of central trope and central film. The text is now written as follows (line 344, page 9):

> *The strategic diagram is widely used in scientometrics, helping visualize the degree of development of a thematic community (cluster) by analyzing the networks of co-words using the keywords of the papers. It is based on two measures:*
>
> - *Callon's centrality: it indicates how well a theme is connected to other themes (external cohesion). It is defined as:* $c = 10 \times \sum e_{kh}$ *with* $k$ *word belonging to the theme and* $h$ *word belonging to another themes.*
> - *Callon's density: it measures the internal strength of a network and it can be defined as* $d = 100\left(\sum e_{ij}/w\right)$*, with* $i$ *and* $j$ *belonging to the theme and* $w$ *being the total number of words in the theme.*
>
> *Moreover, the themes plotted in the strategic diagram are labelled by its central node (referred in the text as central trope or central film, depending on the network), which is the node with the most degree in the theme, formally* $n = \operatorname{argmax}_v(\deg(v))$*. Using the callon's density and centrality it is possible to visualize the communities in four quadrants, whose center is in the position (0.5,0.5). These quadrants indicate if communities are motor (central and developed communities), transversal (basic and general), specialized/peripheral, or emerging/declining. This will help to answer RQ4.*

---

**RV:**

5. In Table 2, the authors should state that data for the period (2000,2020] is not complete.

---

**AA:**

Thanks to the reviewer's feedback we have included the statement in the caption of Table 2. Hence, the caption is written as follows:

> *Descriptive analysis of votes and ratings by periods for all films of the dataset. Notice that the data for the period (2000, 2020] is not complete.*

---

**RV:**

6. In table 4 and 5, the authors should increase font size. Table 4 is not readable. Also, quality of the figures 5,6,8 and 9 is very poor and it is difficult to interpret the results.

---

**AA:**

Font size in tables have been increased to facilitate the reading.

We are aware of the situation of the images, as other reviewers also pointed out the same issue. However, we have scrupulously followed the requirements of the article submission system: we uploaded our high-resolution TIFF images outside the text of the manuscript, using a web form in an additional step of the upload process. The images in the paper have been automatically generated at low resolution by the journal web system and embedded at the end of the PDF. We understand that in the final publication of the article these images will be displayed at higher resolution using our uploaded high-quality versions. We apologise for any inconvenience this may have caused.

---

**RV:**

7. The language needs to be improved since the manuscript contains a number of grammatical errors throughout it (the caption of Fig.1, etc).

---

**AA:**

We want to thank the reviewer suggestion. We have improved the language along the manuscript. Changes have been highlighted in blue.

---

## 2 Reviewer #2

**Legend:**

RV: Reviewer.

AA: Author answers.

---

**RV:**

In this interesting manuscript, authors analyse the distribution and association of tropes in movies, I.e. what they call troposphere. They do this by relying on network-based and scientometrics tools - which makes completely sense, as keywords in papers are like tropes in movies. The manuscript is really interesting and well written. To be honest, I have no major comments on it; for me, it could be published as it is. I'm just adding below a couple of "ideas" (please disregard them if not feasible), and some minor comments.

---

We thank the reviewer for the kind comments and are glad the reviewer found the paper interesting.

---

**RV:**

"Ideas":

Evolution of trope reception. Authors have calculated the relationship between tropes and their reception, i.e. the associated films' popularity. Yet, can it be that this association is temporal dependent? Imagine a film introducing a completely new trope, something never seen before. That movie could be appraised for being really original. Now, if hundreds of movies start using the same trope... that may get boring, and the associated rating may drop. Is there any way of measuring this? (This last part reminds me of the YouTube channel CinemaSins... if you know it, you will surely understand why I'm suggesting this!)

---

**AA:**

The reviewer is right. The *slasher* trope, and its first-person perspective, of the film *Halloween* had never been seen before and that is why this film is so good, even though such tropes are very common these days. We think it is hard to judge when a new trope will produce an improvement. For example, as a viewer's film culture increases, the appearance of repeated tropes can cause to lose interest in a movie they see later. Also, when a novel trope appears in place of a familiar one, it will not always be accepted by the audience. For example, in *The Last Jedi*, several tropes were subverted, which led to the rage of many fans. Therefore, it is difficult to gauge when the appearance (or subversion) of a new trope will be better than using the most common trope. Nevertheless,

it is another very interesting topic to study in future work, and we thanks the reviewer for the idea.

By the way, if the reviewer is interested in the *CinemaSins* channel, we also recommend its not so well known nemesis: *CinemaWins*, which, on the contrary, shows the good parts of films, even from the movies that are reviled by critics.

---

**RV:**

Trope prediction. I would have enjoyed a final (speculative and personal, of course) prediction in the conclusions. For instance, having seen the data, which tropes are going to gain momentum in the future?

---

**AA:**

This is a good idea. We have added the next paragraph in the conclusions (line 706, page 17).

> *As indicated in the results, there are communities in the emerging quadrant that are precisely related to previous adaptations or versions (ItWasHisSled, AdaptationalJerkass and DeconstructiveParody). We believe that this may be due to the large number of remakes and adaptations of other works that are currently being produced, so we speculate that many of new works will also be adapted to the new times in one way or another.*

---

**RV:**

Line 138: "quality and popularity of the films". Is quality a good word here? Because it suggests an objective measure, such as how good a film is from a technical perspective... but it's not clear to me how this can be measured, and if this is actually taken into account in the manuscript.

---

**AA:**

We share the point of view of the reviewer. We have changed the word "quality" and used "rating" instead.

---

**RV:**

Line 257: "For the 100 most voted or rated tropes in genres." Any rationale for choosing 100?

---

**AA:**

We thank the reviewer question. Given the space of the paper, we have considered that 100 is an optimum number to show the most featured tropes.

**RV:**

Algorithm 1. Is this really needed? I mean, anyone working in networks will know how to create a co-ocurrence network… It seems to me that the textual explanation is more than enough.

**AA:**

We appreciate the reviewer question. We took into account the potential public of this paper and we considered to include it for the sake of understandability.

**RV:**

Line 280. “Clustering” may not be the best word here, as in complex network theory this reminds me of “assortativity”... why not just use modularity? Or “community detection”? At least, I would clarify that “clusters” and “communities” are used interchangeably in the paper, and that the former does not refer to transitivity.

**AA:**

We agree with the reviewer. We have changed ”Clustering” (line 280) by “Community detection” (now in line 324, page 8) which is more appropiate in the context. Moreover, we have unified the word “cluster” into “community” in the rest of the text to avoid potential confusions.

**RV:**

Line 517. “overview of which of these tropes may be interesting to develop in the future”. Not really, if I understood this correctly. That is, ascending and declining tropes are in the same quadrant… so, how can they be discriminated? With a time-dependent analysis?

**AA:**

The reviewer is right, the ascending and declining tropes are in the same quadrant, so they are discriminated via a time-dependent analysis as proposed in [3]. In this paper, we do it as shown in Figure 7 of the paper, where all the communities in the quadrant are ascending as the number of films increases by each decade. However, some communities are more noticeable than others such as *ItWasHisSled* or *DeconstructiveParody*.

**RV:**

Line 219: “an specific methodology” `->` “a”
Line 294: “To confirm that there is statistical significance between” I think “relationship” is missing... Same at line 427.
Line 550: “gender”? Or genre?

---

**AA:**

Thank the reviewer for the corrections, the proposed changes have been made.

---

**RV:**

On a side note, I find the abstract a little long. The third paragraph is perfect, but maybe the first two could be merged and eventually synthesised a little? In any case, this is more a question of style (and taste), not of scientific content.

---

**AA:**

Without losing information, these two paragraphs have been merged and now read:

> *Creating a story is a challenging task due to the the complex relations between the parts that make it up, which is why many new stories are built on those cohesive elements or patterns, called tropes that have been shown to work in the past. A tropes is a recurring story-telling device or pattern, or sometimes meta-elements, used by the authors to express ideas that the audience can recognize or relate to, such as the Hero's Journey. Discovering tropes and how they cluster in popular works and doing it at scale to generate new plots may benefit writers. In this paper, we analyze them and use a principled procedure to identify trope combinations, or communities, that could possible be successful. The degree of development of these different communities can help us identify areas that are under-developed and, thus, susceptible to such a type of development.*

We thank the reviewer for this suggestion.

---

# 3 Reviewer #3

**Legend:**

RV: Reviewer.

AA: Author answers.

---

**RV:**

This is an interesting paper studying the plot of stories. While the results are relevant and worthy publishing, some issues should be addrssed before this paper can be considered adequate for publication:

---

**AA:**

We want to thank the reviewer for the and we are very glad that you found the paper interesting.

---

**RV:**

Section of related works could be improved by mentioning similarities and differences with stories and texts modeled as co-occurrence networks. See and mention e.g. doi: 10.1209/0295-5075/114/58005 doi: 10.1016/J.INS.2018.02.047 .

---

**AA:**

We thank the reviewer for these new references. Both of them have been added and commented in the state of the art (line 222, page 6):

> *Amancio [2] proposed the use of co-occurrence networks to study and detect the different entities (characters or locations) and their semantic relationships, which appear in different novels. A topological analysis was applied to obtain patterns that were not possible to detect using classical methods. Another application of the use of complex networks is the identification of the meaning of words with multiple meanings. The work [10] describes a process based on a bipartite network model that outperforms widely used machine learning methods to deal with this issue. However, as stated before, these works rely on the analysis of whole texts to obtain the relationships between entities, whereas our work is based on the mere occurrence of tropes in the different films.*

---

**RV:**

some steps in the methodology should be clarified. For example, adjustments of the dataset is not informative. This could mean anything.

---

**AA:**

We agree with the reviewer, we have changed the name to “Dataset preprocessing”.

---

**RV:**

Methodological steps should be presented in a better way. For example, performance analysis might include performance measures and visualization. Alternatively, clustering analysis could include items 6, 7 and 8.

---

**AA:**

We want to thank the reviewer for the comment. The steps explained in this manuscript, are described in a similar way in other published works [1]. Nonetheless, some names may lead to confusion so we have changed them and modified the “Adjustments of the dataset” step as follows (line 277, page 7):

> *Dataset pre-processing: in this study, two datasets are considered:*
>
> - *The dataset obtained from the data acquisition phase. In the rest of the text it will be referred as $D_f$.*

---

**RV:**

A descriptive analysis of the dataset could be provided when presenting the dataset. This can not be considered a main result.

---

**AA:**

Thank you for your comment. We present the descriptive analysis in a different section, Experiments and results, and it is used to solve the first research question (RQ1).

---

**RV:**

It is not clear why Leiden algorithm have been used for this analysis. I suggest using SBM to check the robustness of results.

---

**AA:**

Thank you for your comment. In the literature [5], various algorithms have been proposed to deal with community detection in networks, in fact, methods such as blockmodeling, spectral clustering, or modularity-based clustering among others are very adequate to address the problem. It is also known that there is no algorithm better than other but it depends on the needs of the study. In our case we have used the Leiden algorithm because its features suit perfectly with the problem we are addressing. The features that lead us to choose the Leiden algorithm are:

1. It ensures well connected communities.

2. All the communities are subset optimal, which ensures that all subsets of the community are locally optimally assigned and cannot be moved to a different community.

According to the above arguments, we think that Leiden algorithm, which is modularity-based, is a good option but other algorithms would be valid as well.

Concerning the robustness, we know that algorithms based on modularity could give different results. In our case, we have executed of the Leiden algorithm with the `n_iterations` input parameter equals `-1`, so it is run until an iteration in which there is no improvement. Therefore, we have checked the robustness of our results in two different ways:

- We ran the algorithm multiple times and no substantial differences in the communities were found.

- The detection of communities have been examined by domain experts, who agree with the results.

In addition, we want to point out that the Leiden algorithm [14], published on the journal *Scientific Reports* (Nature Publishing Group) in 2019, has 94 citations in the Web of Science database, and among them, there are important applications to other fields such as:

- "Antibiotics-Driven Gut Microbiome Perturbation Alters Immunity to Vaccines in Humans" [8], published on *Cell* journal, which is the first journal in terms of impact factor in the area of Biochemistry & Molecular Biology, as well as the second one in the area of Cell Biology.

- "Identification of a T follicular helper cell subset that drives anaphylactic IgE" [7], published on *Science* journal, which is the second journal ranked in Multidisciplinary Sciences.

- "Assessing police topological eficiency in a major sting operation on the dark web" [4], published on *Scientific Reports*, which has a great impact factor in the area of Multidisciplinary Sciences.

Furthermore, the Centre for Science and Technology Studies (CWTS) based in Leiden, has collaborated in the development of Citation Topics in InCites (Clarivate) [11]. Citation Topics is a new document-level classification scheme for InCites Benchmarking & Analytics™, and it has been developed with the power of the Leiden community detection algorithm as commented in the Global ISI Reports 2021 [12].

---

**RV:**

Could your methodology be a used in a practical contexts, e.g. to make any type of prediction.

---

**AA:**

The practical applications of this paper may be of interest to a wide range of professionals. For example, film historians can use our methodology to study how tropes have evolved over time, or the cultural differences from films of different countries. Moreover, understanding how tropes relate to each other in a quantitative way can also benefit researchers in other fields, such as automatic narrative generation. We have therefore added the following paragraph to the paper (line 167, page 5):

> *In general, we think that this methodology can be useful since it gives us a quantitative approach to the analysis of narrative devices, which can be easily turned into a methodology for generation of narratives, as a well as a for critical analysis of these systematized narratives in the past. In this sense, we think it can benefit videogame creators, as well as media history scholars.*

---

**RV:**

It is not clear why word analysis mentioned here is related specifically to bibliometrics analysis. co-occurrence analysis have been used in many contexts other than scientometrics analysis, including e.g. in traditional text analysis: doi: 10.1140/epjb/e2008-00206-x doi: 10.1016/j.physa.2018.03.013

---

**AA:**

The reviewer is right. We related word analysis specifically to bibliometrics analysis because we have a bibliometrics background. Nonetheless, we agree with the comment, and included the references given by the reviewer in the related work as follows (line 209, page 5):

> *Moreover, word analysis has been applied in other contexts such as traditional text analysis. For instance, Herrera et al. [9] proposed a detector, which is based on unsupervised statistical methods, for*

*detecting keywords in texts. Other application to traditional text analysis is presented by Tohalino et al. [13], in which a multilayer network is used to address the extractive multidocument summarization task. However, in our study we do not analyze corpus of text, but the relationship between movies and the tropes they appear in them, and vice versa.*

---

**RV:**

Figures are in low quality. I suggest using a vector format. I can not read most of labels in the larger network.

---

**AA:**

We are aware of the situation, as other reviewers also pointed out the same issue. However, we have scrupulously followed the requirements of the article submission system: we uploaded our high-resolution TIFF images outside the text of the manuscript, using a web form in an additional step of the upload process. The images in the paper have been automatically generated at low resolution by the journal web system and embedded at the end of the PDF. We understand that in the final publication of the article these images will be displayed at higher resolution using our uploaded high-quality versions. We apologise for any inconvenience this may have caused.

---

## 4 Reviewer #4

**Legend:**

RV: Reviewer.

AA: Author answers.

---

**RV:**

This paper investigates the nature of tropes in films using crowd-sourced data as its primary source material. The paper offers simple summary statistics of the data before applying statistical methods to develop network views in the spaces of tropes, genres and films.

First, a caveat. This reviewer is a physicist with a background in statistics of large-scale cosmic structure and no experience in reviewing Film, Television and Media (FTVM) literature. Like most adult humans, I do have some experience watching movies and the Simpsons.

**AA:**

We want to thank the reviewer for the valuable comments.

**RV:**

The authors do not clear who is the primary audience for this work. Is this an academic work aimed at FTVM faculty? A practical work aimed at studio executives and/or independent filmmakers? An exploratory work aimed primarily at burnishing the reputations of the authors among a burgeoning group of trope analysts? The text is unclear.

In my opinion, the present draft has too many deficiencies to warrant publication as is. A revised version could be acceptable if it addresses these key, enumerated issues.

**AA:**

Studio executives and/or independent filmmakers are not known to read scholarly papers. We'd be much honored if they did, but they will probably not. This is, quite obviously, an academic work, aimed at the readers of this journal; it's going to be openly published, so its audience might potentially quite wide. We really don't know there's such thing as a group of trope analysts, but we certainly hope that if the paper is published (with your approval), our reputation is not tarnished.

However, I think the specific audience is beside the point of the paper (and it should certainly be for most papers we have written in the past and will write in the future). The objective of the paper is to provide a way to analyze story patterns at scale, using as source a crowdsourced platform. As such, it can benefit historians, the FTVM faculty you mention, as well as anyone wanting to generate stories at scale, such as video game producers. You certainly have a valid point indicating that this has not been sufficiently clarified in the paper, which is why we have added this (line 167, page 5):

> *In general, we think that this methodology can be useful since it gives us a quantitative approach to the analysis of narrative devices, which can be easily turned into a methodology for generation of narratives, as a well as a for critical analysis of these systematized narratives in the past. In this sense, we think it can benefit video game creators, as well as media history scholars.*

We will also do our best to address your other points below.

**RV:**

1) The tone of the work is too authoritative given its exploratory nature. For example, the abstract states that "Developing plots or narrations requires understanding the relationships between tropes in existing works,. . . " This is an overstatement; there is no such requirement. Line 11 of the main text claims we will "understand" human behaviors from crowdsourced data when it would be better to say that such sources offer a "window toward understanding".

**AA:**

The first two paragraphs of the abstract have been rewritten, they now read

> *Creating a story is a challenging task due to the the complex relations between the parts that make it up, which is why many new stories are built on those cohesive elements or patterns, called tropes that have been shown to work in the past. A trope is a recurring storytelling device or pattern, or sometimes a meta-element, used by the authors to express ideas that the audience can recognize or relate to, such as the Hero's Journey. Discovering tropes and how they cluster in popular works and doing it at scale to generate new plots may benefit writers; in this paper, we analyze them and use a principled procedure to identify trope combinations, or communities, that could possible be successful. The degree of development of these different communities can help us identify areas that are under-developed and, thus, susceptible to such a type of development.*

The second request has been changed in the way requested. Thanks for the suggestion.

**RV:**

2) The figures and table are often difficult to read, particularly in printed form. Figures 6 and 8 are particular examples in which important labels are hard to make out. Tables 4 and 6 have very small font sizes.

**AA:**

Font size in tables have been increased.

We are aware of the situation of the Figures, as other reviewers also pointed out the same issue. However, we have scrupulously followed the requirements of the article submission system: we uploaded our high-resolution TIFF images outside the text of the manuscript, using a web form in an additional step of the upload process. The images in the paper have been automatically generated at low resolution by the journal web system and embedded at the end of the PDF. We understand that in the final publication of the article these images

will be displayed at higher resolution using our uploaded high-quality versions. We apologise for any inconvenience this may have caused.

---

**RV:**

3) The authors should do more to critically address the quality of their data. Should all of the nearly 26,000 tropes be considered as independent? Is popularity on IMdB different from that on other ratings platforms? The authors did not provide rationale for why the specific data sets (TV Tropes.org; IMDb) were used over, or in exclusion of, other databases such as allthetropes.com; Metacritic, Google User Reviews: Rotten Tomatoes. While the authors acknowledge bias in database used, cross referencing across multiple review databases would potentially produce different complex network results.

---

**AA:**

We have used IMDB because it allows you to use an API in a straightforward way and get batch information for all their films at once. In the case of RottenTomatoes and MetaCritic, requests have to be made individually per film, and there is also a limit per user, making the download of all the data unfeasible in a reasonable time. In addition, the metadata of the retrieved films is more limited.

Regarding the downloading of tropes, we have used TVTropes instead of AllTheTropes, because the latter is a fork of the former, containing practically the same information, but TVTropes being more widely used and known.

Nevertheless, the idea proposed by the reviewer is interesting for future studies, so it has been mentioned in future work at the end of the paper (line 733, page 18).

> *This issue can be addressed by adding other sources of data such as RottenTomatoes*[1] *or MetaCritic*[2]*.*

---

**RV:**

4) Language in the methodology section is awkward and contains grammatical errors that negatively affect readability. Would recommend editing for English clarity.

---

**AA:**

Thank to the reviewer comment. We have improved the language of the paper.

---

[1] https://www.rottentomatoes.com/

[2] http://www.metacritic.com

**RV:**

Overall I was hoping to learn something new from this paper but came away mostly confused. The authors would do well to embrace the "Less is more" philosophy here. A study confined to the top-100 most frequent tropes across the top 10 genres would be easier to digest compared to one that tackles everything all at once.

---

**AA:**

We understand what the reviewer is suggesting, and hope this new version is less confusing than the initial one, after addressing all concerns. However, we would like to point out that the paper is mainly methodological and is not so much concerned with creating (forcibly ephemeral) rankings, than with proposing a methodology to analyze how tropes cluster together in movies, and how this can be used as an inspiration for future naration topics.

We still think that the paper works as a whole, since we are trying to address different research questions and all parts work together towards the answer. We will try, however, to be if not briefer, at least more direct in getting through to the prospective reader. We will try to address the rest of your concerns below.

---

**RV:**

Figures 1 and 3 would benefit from a log-log display (with special treatment for zeros), making the power-law nature of the frequency distributions more apparent.

---

**AA:**

We appreciate the reviewer suggestion. We have updated the figures to the requested log-log display, they are shown in Figures 1 and 2. Moreover, we have rewritten the captions to improve the awkward English as other reviewers have pointed out. Additionally, we have referred to the Figures 1 and 2 as log-normal distributions.

---

**RV:**

Lines 61,2: Sample biases must be understood to contextualize the understanding derived from network analysis.

---

**AA:**

As indicated in [6], there is clearly a bias in the sample that we are using: more popular movies will attract more attention from the public, and more tropes will be reported, and and the time bias is also clear, with current movies getting more tropes than old ones. This is inherent in the source we are using,

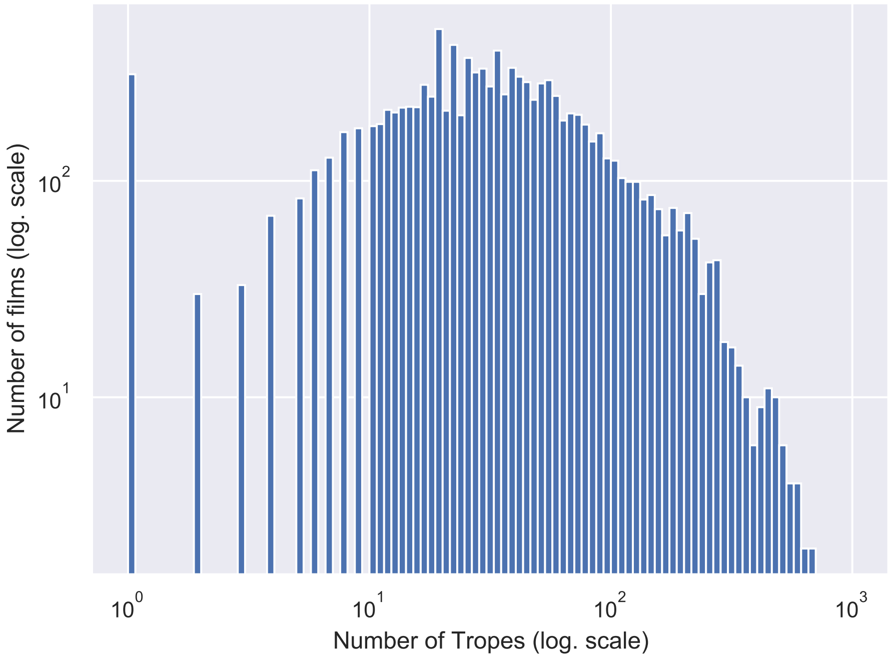


Figure 1: Distribution of tropes in films (log-log scale is applied, zeros were treated adding one to all the values).

and there's relatively little we can do to overcome this. We have added, however, a clarification about this bias, clarifying that, while it would obviously be a problem in a rigorous and precise historic and quantitative analysis, this is not really our objective. This text has been added to the paper to clarify this point (line 72, page 3).

> *In this endeavor we should not lose sight of the fact that the source that we are using is biased towards popular and recent films, so some stories that have not reached popularity for some reason (minority language, for instance) or that were released a long time ago might have a winning trope combination that is not reflected in our study. Besides, this bias is not constant, so we can't really affirm that some specific genre or kind of movie has been left out (or included) uniformly across the data we have analyzed.*
>
> *Fortunately, this bias aligns with our objective, which is narrative generation in the present sense, as well as the creation of narratives that might be cohesive. We will assume that a part of what makes a movie popular is the cohesiveness of the tropes they employ, or how they work together with each other. This means that our results will probably not be affected by this bias, that will nonetheless have to be*

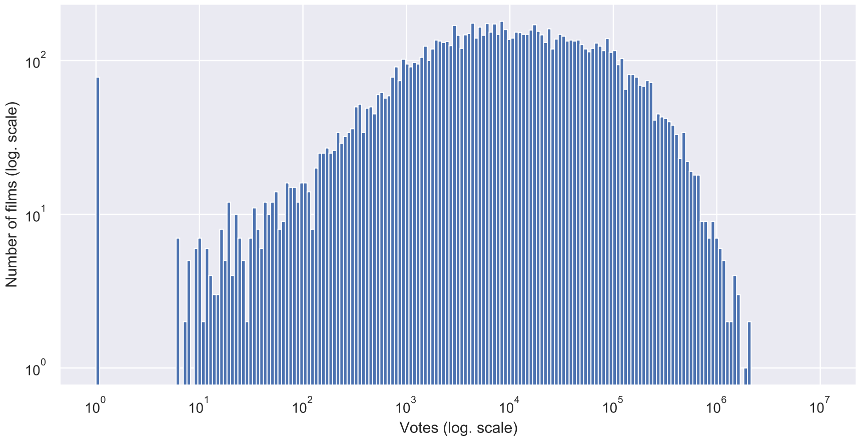


Figure 2: Distribution of votes (popularity) in films (log-log scale is applied, zeros were treated adding one to all the values).

*taken into account when working towards a different objective.*

---

**RV:**

Line 80: Cite needed for Leiden algorithm.

---

**AA:**

As the reviewer pointed out, we included the cite.

---

**RV:**

Line 114: "actual one" : how is the true trope nature of a film defined here?

---

**AA:**

We have rewritten the phrase, as we were referring to the actual set of tropes in a film, with respect to the set detected by users on the website.

---

**RV:**

Line 133: The community labels used here should be defined for the benefit of those unfamiliar with these terms. And/or a cite added.

---

**AA:**

Thank you for your comment. We have cited the paper where the community labels are explained.

---

**RV:**

Line 281: A brief explanation of Jaccard index is warranted here. And/or move up explanatory equation.

---

**AA:**

Thank you for your comment. We have included a brief explanation of Jaccard index in the overlapping analysis step of the methodology. The included text is the following (line 292, page 7):

> *A well-known measure of overlapping is the Jaccard index, which measures the similarity between two sets, and it is defined as the size of the intersection divided by the size of the union of the sets given.*

---

**RV:**

Lines 439-465: Table 4 is exasperating, nearly impossible to read. The description of the results here is helpful, but the fact that the Shout Out trope is nearly universal suggests that this trope should be considered separately from the others. Again, less could be more here.

---

**AA:**

Reviewer is right. We have increased the font size for these tables. Quite clearly, ShoutOut, which is essentially a reference to other films, characters or popular culture, is a staple of modern films, and it really stands out among the rest. However, our methodology does not allow to consider it separately. It would be interesting, however, to study how the inherently networked nature of this trope makes it so popular, and literally so central. That is, however, beyond the focus of this paper.

---

**RV:**

Line 513: grammar Lines 233,4,7: Examples of awkward English (Point 4 above).
Line 122: who is the "us" being referred to here?
Line 106: "solve" -> "address"
Line 550: gender -> genre

---

**AA:**

We changed all the minor corrections proposed by the reviewer. Regarding the awkward English, we have done the following:

1. Phrase in Line 513 has been rewritten (now in line 565, page 14):

   > *In addition, there has been an apparent increase in the number of films from some communities in recent years.*

2. Lines 233-234. The English has been reviewed and we have extended the explanation for the sake of clarity. Therefore the paragraph has been changed to (now in line 267, page 7):

   > *Preliminary analysis of the dataset: To get an overview of the dataset, a descriptive analysis is proposed. The analysis includes the standard deviation, mean, minimum, maximum and the first, second and third quartiles of votes, ratings and tropes. Moreover, a visualization of the distributions of the number of tropes, ratings and votes is shown. Furthermore, a line chart with the films per year is presented. Finally, the votes and ratings in periods of two decades from 1880 to 2020 are analysed as well as the distribution of genres in the dataset.*
   >
   > *All the previous analysis will help us to detect whether some biases exist or not. Additionally, the analysis will give information to answer the first research question (RQ1).*

3. Line 237. We have changed the sentence to (now in line 278, page 7):

   > *The dataset obtained from the data acquisition phase. In the rest of the text it will be referred as $D_f$.*

Moreover, concerning the line 122, we removed the "us" so the sentence now is written as follows (line 134, page 4):

> *generally genre (or combination thereof) comes before the plot itself, and understanding how tropes shape genres or the other way out will help to generate better stories.*

---

**RV:**

Reference 1 is incomplete

---

**AA:**

Thanks to the reviewer's feedback, we updated the reference 1. Now, it is written as follows:

*Bellanova R, González Fuster G. No (Big) Data, no fiction? Thinking surveillance with/against Netflix. In: Rudinow A, Schneider I, Green N, ors. The Politics and Policies of Big Data: Big Data Big Brother? Routledge; 2018. p. 227–246.*

# References


[1] Laura Alcaide–Muñoz, Manuel Pedro Rodríguez–Bolívar, Manuel Jesús Cobo, and Enrique Herrera–Viedma. Analysing the scientific evolution of e-government using a science mapping approach. *Government Information Quarterly*, 34(3):545 – 555, 2017.

[2] Diego Raphael Amancio. Network analysis of named entity co-occurrences in written texts. *EPL (Europhysics Letters)*, 114(5):58005, jun 2016.

[3] M J Cobo, A G López-Herrera, E Herrera-Viedma, and F Herrera. An approach for detecting, quantifying, and visualizing the evolution of a research field: A practical application to the Fuzzy Sets Theory field. *Journal of Informetrics*, 5(1):146–166, 2011.

[4] Bruno Requiao da Cunha, Padraig MacCarron, Jean Fernando Passold, Luiz Walmocyr dos Santos, Jr., Kleber A. Oliveira, and James P. Gleeson. Assessing police topological efficiency in a major sting operation on the dark web. *SCIENTIFIC REPORTS*, 10(1), JAN 9 2020.

[5] Santo Fortunato. Community detection in graphs. *Physics Reports*, 486(3):75 – 174, 2010.

[6] Rubén Héctor García-Ortega, Pablo García-Sánchez, and Juan Julián Merelo Guervós. Tropes in films: an initial analysis. *CoRR*, abs/2006.05380, 2020.

[7] Uthaman Gowthaman, Jennifer S. Chen, Biyan Zhang, William F. Flynn, Yisi Lu, Wenzhi Song, Julie Joseph, Jake A. Gertie, Lan Xu, Magalie A. Collet, Jessica D. S. Grassmann, Tregony Simoneau, David Chiang, M. Cecilia Berin, Joseph E. Craft, Jason S. Weinstein, Adam Williams, and Stephanie C. Eisenbarth. Identification of a T follicular helper cell subset that drives anaphylactic IgE. *SCIENCE*, 365(6456):883+, AUG 30 2019.

[8] Thomas Hagan, Mario Cortese, Nadine Rouphael, Carolyn Boudreau, Caitlin Linde, Mohan S. Maddur, Jishnu Das, Hong Wang, Jenna Guthmiller, Nai-Ying Zheng, Min Huang, Amit A. Uphadhyay, Luiz Gardinassi, Caroline Petitdemange, Michele Paine McCullough, Sara Jo Johnson, Kiran Gill, Barbara Cervasi, Jun Zou, Alexis Bretin, Megan Hahn, Andrew T. Gewirtz, Steve E. Bosinger, Patrick C. Wilson, Shuzhao Li, Galit Alter, Surender Khurana, Hana Golding, and Bali Pulendran. Antibiotics-Driven Gut Microbiome Perturbation Alters Immunity to Vaccines in Humans. *CELL*, 178(6):1313+, SEP 5 2019.

[9] J. P. Herrera and P. A. Pury. Statistical keyword detection in literary corpora. *European Physical Journal B*, 2008.

[10] Edilson Anselmo Corrêa Júnior, Alneu de Andrade Lopes, and Diego R. Amancio. Word sense disambiguation: A complex network approach. *Inf. Sci.*, 442-443:103–113, 2018.

[11] Ingo Lütkebohle. Introducing Citation Topics in InCites. `https://clarivate.com/webofsciencegroup/article/introducing-citation-topics/`, 2020. [Online; accessed 03-02-2021].

[12] Martin Szomszor, Jonathan Adams, David A. Pendlebury, and Gordon Rogers. Data categorization: understanding choices and outcomes. `https://clarivate.com/webofsciencegroup/campaigns/data-categorization-understanding-choices-and-outcomes/`, 2021. [Online; accessed 17-02-2021].

[13] Jorge V. Tohalino and Diego R. Amancio. Extractive multi-document summarization using multilayer networks. *Physica A: Statistical Mechanics and its Applications*, 2018.

[14] V. A. Traag, L. Waltman, and N. J. van Eck. From Louvain to Leiden: guaranteeing well-connected communities. *Scientific Reports*, 9(1):5233, 2019.
